# Leiomyosarcoma of the inferior vena cava: a case report of a challenging diagnosis of abdominal pain

**DOI:** 10.1590/1677-5449.202501152

**Published:** 2026-03-23

**Authors:** Amélia Gaspar, Matilde Vala Esteves, Filipe Santos Leal, Clarisse Calça Coelho, Cristina Costa Silva, João Arcanjo

**Affiliations:** 1 USF Mondego - Unidade Local de Saúde de Coimbra – ULS Coimbra, Coimbra, Portugal.

**Keywords:** leiomyosarcoma, vena cava, inferior, abdominal pain, diagnosis, differential, leiomiossarcoma, veia cava inferior, dor abdominal, diagnóstico diferencial

## Abstract

Leiomyosarcoma of the inferior vena cava (IVC-LMS) is a rare malignant tumor, accounting for approximately 2% of all leiomyosarcomas, and predominantly affecting women over 50 years of age. Patients are often asymptomatic or present with nonspecific abdominal pain, which can hinder and delay diagnosis. We report the case of an 80–year–old male patient with a 3-month history of nonspecific abdominal pain. The diagnosis was established only intraoperatively, when the smooth muscle neoplasm initially suspected to originate from the duodenum was identified as an IVC-LMS. The patient underwent partial resection of the IVC with prosthetic interposition, followed by adjuvant chemoradiotherapy. Complete surgical resection remains the gold standard treatment of IVC-LMS and the only approach with curative potential. Prognosis largely depends on the stage at diagnosis.

## Resumo

O leiomiossarcoma da veia cava inferior (LMS-VCI) é um tumor maligno raro, responsável por aproximadamente 2% de todos os LMSs, que afeta principalmente mulheres com mais de 50 anos de idade. Os pacientes são muitas vezes assintomáticos ou apresentam dor abdominal inespecífica, o que dificulta e atrasa o diagnóstico. Este caso descreve um paciente do sexo masculino, de 80 anos de idade, com histórico de dor abdominal inespecífica há três meses. O diagnóstico foi estabelecido apenas no intraoperatório, revelando que a neoplasia do músculo liso, inicialmente suspeita de origem duodenal, era na realidade um LMS-VCI. O paciente foi submetido a resseção parcial da VCI com interposição de prótese, seguida de quimiorradioterapia adjuvante. O tratamento padrão-ouro para o LMS-VCI, e o único com potencial curativo, é a resseção cirúrgica completa. Seu prognóstico depende da fase em que é diagnosticado.

## INTRODUCTION

Leiomyosarcoma of the inferior vena cava (IVC-LMS) is a rare malignant tumor originating from the smooth muscle cells of the vein's tunica media and accounts for approximately 2% of all leiomyosarcomas.^1-3^ This tumor predominantly affects women over 50 years of age.^1,3,4^ Patients are often asymptomatic or present with nonspecific abdominal symptoms, particularly in cases of large tumors, which can complicate and mislead diagnosis.^1,3-6^ Due to its rarity, data regarding prognosis and therapeutic options remain limited, and surgical resection is considered the only potentially curative treatment.^2,4,6,7^

We present the case of an 80–year–old male patient with IVC-LMS that was preoperatively presumed to be a smooth muscle neoplasm of the duodenum.

## CASE PRESENTATION

This case describes an 80–year–old male patient with a medical history of systemic arterial hypertension and benign prostatic hyperplasia, treated with bisoprolol 5 mg and lansoprazole 15 mg. He had no history of tobacco or alcohol use.

In October 2021, the patient developed abdominal pain in the right iliac fossa, which he associated with changes in bowel habits, prompting a colonoscopy. One month later, he returned with epigastric pain, dyspepsia, and nausea, leading to the request for an upper gastrointestinal endoscopy. The examination revealed a duodenal polyp, for which endoscopic excision was indicated, and the patient was subsequently referred to the Gastroenterology department.

In January 2022, he returned for consultation with persistent pain, now localized to the right flank and radiating to the right iliac fossa, accompanied by anorexia and nausea. An abdominal ultrasound was requested and revealed the following findings: “In the region of the transition from the right flank to the right iliac fossa, a hypoechoic mass is observed, apparently involving the ascending colon, measuring 70 x 69.8 x 41.7 mm (longitudinal × transverse × anteroposterior), for which further evaluation with colonoscopy and computed tomography (CT) was recommended.” At that time, physical examination was unremarkable, except for a weight loss of 3 kg over 4 months.

**Figure 1:**
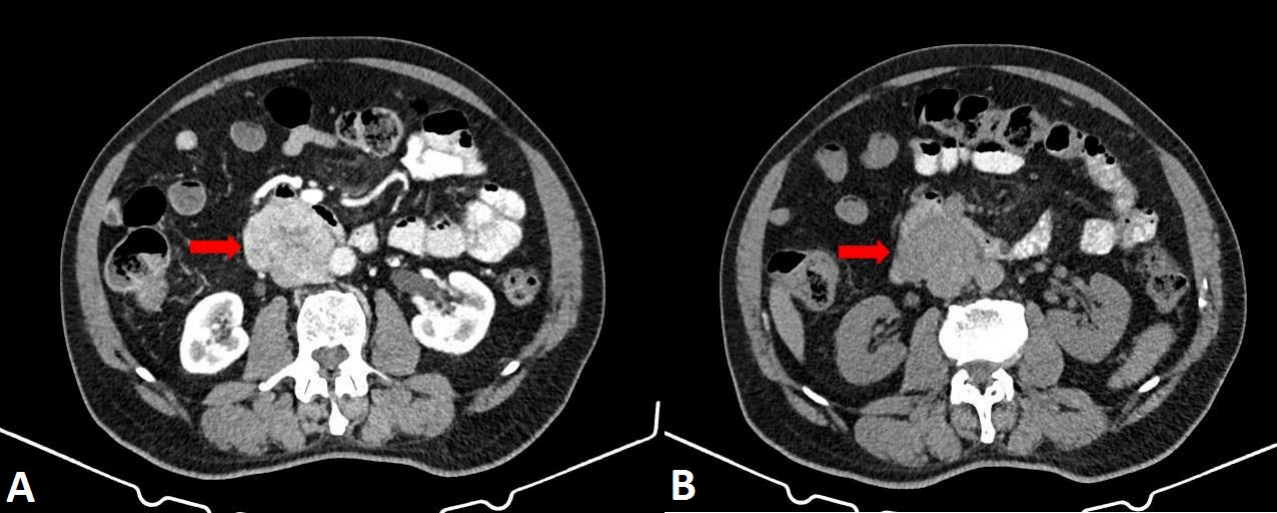
Abdominal computed tomography scan, axial views. Red arrows indicate the large (70 x 60 x 50 mm; longitudinal x transverse x anteroposterior) solid, heterogeneous, expansile mass with lobulated contours (images A and B), located in the median and right paramedian retroperitoneal region, adjacent to the posterior and inferior wall of the third portion of the duodenum, with no clear cleavage plane from the inferior vena cava.

**Figure 2:**
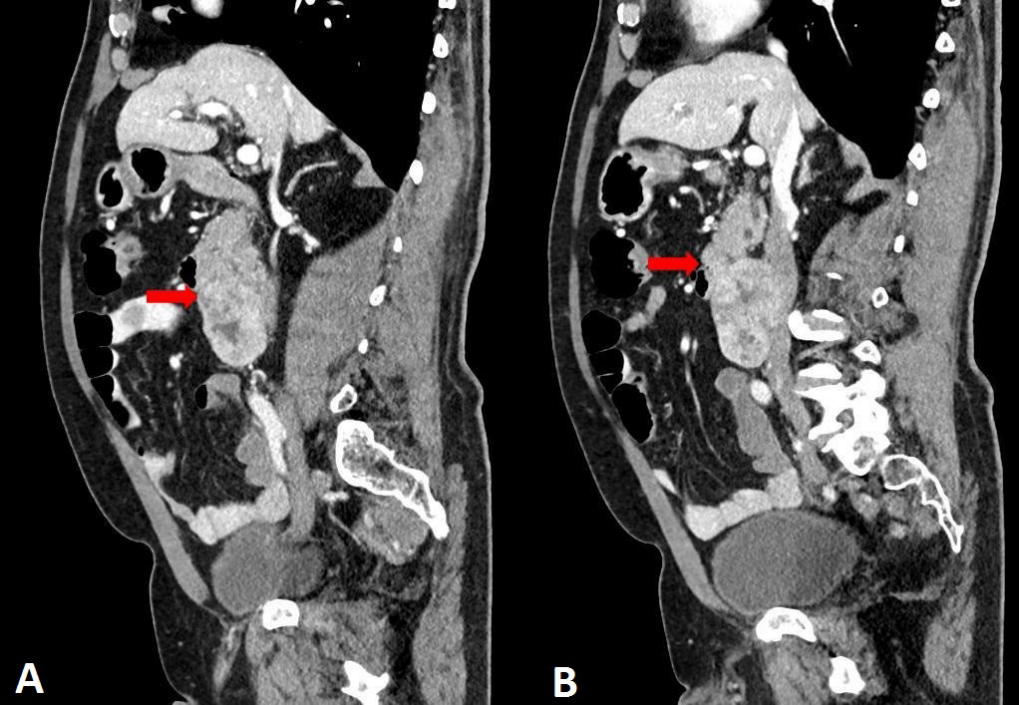
Abdominal computed tomography scan, sagittal views. Red arrows demonstrate a large retroperitoneal mass (images A and B) located inferior to the uncinate process of the pancreas, adjacent to the posterior wall of the third portion of the duodenum (causing anterior deviation), and in close proximity to the right lateral wall of the aorta, with no clear cleavage plane from the inferior vena cava.

An abdominal CT scan was ordered for further evaluation and revealed the following findings: “In the median and right paramedian retroperitoneal region, inferior to the uncinate process of the pancreas (without a discernible cleavage plane) and the duodenal arch, adjacent to the posterior and inferior wall of the third portion of the duodenum, causing anterior displacement, there is a large (70 x 60 x 50 mm; longitudinal × transverse × anteroposterior) solid, heterogeneous, expansile mass with lobulated contours and intense heterogeneous contrast enhancement, containing areas of lower attenuation (suggestive of necrosis). Across the different phases, including the venous phase, the lesion cannot be clearly distinguished from a segment (40 mm longitudinally) of the IVC, raising the possibility of partial intraluminal involvement, partial obliteration, or extrinsic compression. The IVC demonstrates normal caliber both upstream and downstream, as do the iliac veins. The mass is also adjacent to the right lateral wall of the aorta, with partial loss of a cleavage plane along its longitudinal extent. Differential diagnosis considerations include sarcoma, lymphoproliferative disease, or a mass originating from the uncinate process of the pancreas; histopathological evaluation is recommended. No apparent lymphadenomegaly or free peritoneal effusion was identified.” (Figures 1 and 2).

The patient was referred for a surgical consultation and, in March, underwent an endoscopic ultrasound–guided biopsy, which revealed the following: “Smooth muscle neoplasm of the duodenum at the level of D3, with an insufficient sample to assess malignant potential.”

**Figure 3:**
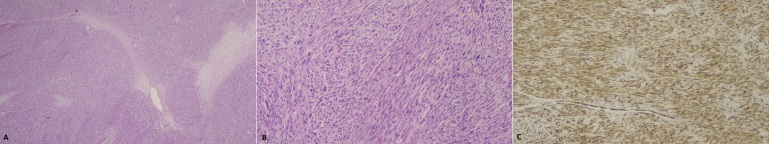
(A) Hematoxylin and eosin (H&E)–stained histological section at x20 magnification, showing a spindle-cell neoplasm with a fascicular growth pattern and moderate cellularity; (B) H&E–stained histological section at x100 magnification, highlighting the cytological features of the spindle cells, including elongated nuclei and eosinophilic cytoplasm, consistent with smooth muscle differentiation; (C) Immunohistochemical staining for caldesmon at x100 magnification, demonstrating strong and diffuse cytoplasmic positivity, supporting smooth muscle differentiation and confirming the diagnosis of leiomyosarcoma.

The patient was electively admitted in May for surgical resection with a preoperative diagnosis of duodenal neoplasm. Intraoperatively, however, a clear cleavage plane separating from the duodenum was identified, indicating an IVC-dependent lesion. This finding led to partial resection of the IVC with interposition of a Dacron graft. Histopathological analysis of the surgical specimen revealed the following findings: “1. Grade 1 IVC-LMS, measuring 7.5 cm in greatest dimension; perineural and intravascular invasion; involvement of surgical margins. 2. peritumoral tissue involved by LMS. TNM classification: T2Nx (AJCC, eight edition); Stage IB; resection status R1” (Figure 3).

Following discussion at a multidisciplinary team meeting, the patient initiated adjuvant chemoradiotherapy. He received external radiotherapy to the surgical bed with a total dose of 50.4 Gy et28 fractions over 5.5 weeks, from July to August 2022, without significant treatment–related adverse events. The patient was followed in the radiation oncology clinic until November 2022 and in the general surgery clinic until December 2024. At present, he is receiving monthly chemotherapy treatment with trabectedin, which has been ongoing since August 2022 with good tolerance, and remains under follow-up in the medical oncology clinic.

## DISCUSSION

This case highlights the diagnostic challenges associated with persistent abdominal pain. We emphasize the importance of considering less common etiologies (particularly IVC-LMS), as early diagnosis is crucial for achieving favorable outcomes, including the possibility of cure and improved survival rates.^6,7^ Due to its retroperitoneal location, symptoms often manifest at a more advanced stage of disease, primarily as a result of compression of adjacent structures.^1,6,7^ Combined with its rarity and nonspecific clinical presentation, diagnosis is frequently delayed, contributing to a poor prognosis.^1,5,7^ Metastasis occurs predominantly via hematogenous dissemination, affecting mainly the lungs, liver, and bones.^2,6^

Recent advances in diagnostic imaging have enabled earlier detection, improving prognosis.^6^ Ultrasound is usually the first-line imaging modality, but its findings are nonspecific, often suggesting masses of various etiologies and limiting accurate topographic localization.^3^ Computed tomography angiography (CTA) is considered the gold standard for diagnosis, as it allows precise localization of the tumor within the IVC, delineates its relationship with adjacent structures, assesses intraluminal involvement, and facilitates surgical planning.^1,3^ Magnetic resonance imaging may further aid in defining tumor extent and resectability.^1,5^ Histological confirmation is required to establish a definitive diagnosis.^2^

As a rare entity, IVC-LMS may not be initially considered in the differential diagnosis. Although CT imaging assists in defining tumor location and guiding surgical planning, its proximity to adjacent structures can lead to misdiagnosis. In the present case, the diagnosis became evident only intraoperatively, as the preoperative suspicion was a smooth muscle neoplasm of the duodenum.

With respect to treatment, complete surgical resection with tumor-free margins, when feasible, remains the gold standard and may require IVC reconstruction, depending on tumor extent.^3-6^ The role of neoadjuvant and adjuvant treatment is controversial; however, both approaches may be beneficial in borderline unresectable tumors to reduce tumor size and improve resectability.^2-4,6,7^ Given its complexity, management of IVC-LMS should be discussed within a multidisciplinary team.^1,2^

Postoperative follow-up can be performed using CTA.^3^ IVC-LMS recurrence occurs more frequently at the local rather than at the systemic level.^3^ Reported 5- and 10-year overall survival rates after surgical resection with tumor-free margins range from approximately 49% to 68% and from 29% to 47%, respectively.^3,4,6,7^

The present case underscores the importance of considering uncommon etiologies in patients with persistent abdominal pain and highlights the need for a multidisciplinary approach to enhance diagnostic accuracy and therapeutic decision-making, thereby improving the likelihood of a favorable outcome.

## Data Availability

Data sharing is not applicable to this article as no data were generated or analyzed.
